# Tranexamic Acid Use in a Patient with a Life-threatening Bleed Exacerbated by Coagulopathy Due to an Aortic Aneurysm with an Endoleak: A Case Report

**DOI:** 10.7759/cureus.5486

**Published:** 2019-08-26

**Authors:** Ashley Werbin, Allen Fong, George Shahin, Aaron Henderson, Luke Surry

**Affiliations:** 1 Emergency Medicine, San Antonio Military Medical Center, San Antonio, USA; 2 Internal Medicine, San Antonio Uniformed Services Health Education Consortium, San Antonio, USA; 3 Internal Medicine: Hematology / Oncology, San Antonio Uniformed Services Health Education Consortium, San Antonio, USA; 4 Internal Medicine: Hematology / Oncology, Eglin Air Force Base Hospital, Florida, USA

**Keywords:** tranexamic acid (txa), disseminated intravascular coagulopathy (dic), vascular endoleak, non-operative, medical management

## Abstract

Tranexamic acid (TXA) is an anti-fibrinolytic agent that inhibits plasminogen activation by binding to its lysine receptor sites and preventing its conversion to plasmin. It stabilizes clots to reduce bleeding and has been used in the setting of trauma, heavy menstrual bleeding, and hematologic malignancies. To our knowledge, there is no mention in the literature of medical management with TXA to treat a life-threatening hemorrhage in the setting of non-operative, endoleakage-induced, chronic disseminated intravascular coagulation (DIC). This case report summarizes the successful use of TXA in a patient with DIC secondary to multiple aortic aneurysms and endoleakage in an effort to stop the expansion of a life-threatening gluteal hematoma not amenable to surgical or vascular intervention.

## Introduction

Tranexamic acid (TXA) is an anti-fibrinolytic agent that inhibits plasminogen activation by binding to its lysine receptor sites and preventing its conversion to plasmin [1]. It stabilizes clots to reduce bleeding and has been used in the setting of trauma, heavy menstrual bleeding, and hematologic malignancies [1]. To our knowledge, there is no mention in the literature of medical management with TXA to treat a life-threatening hemorrhage in the setting of non-operative, endoleakage-induced chronic disseminated intravascular coagulation (DIC).

## Case presentation

**Figure 1:**
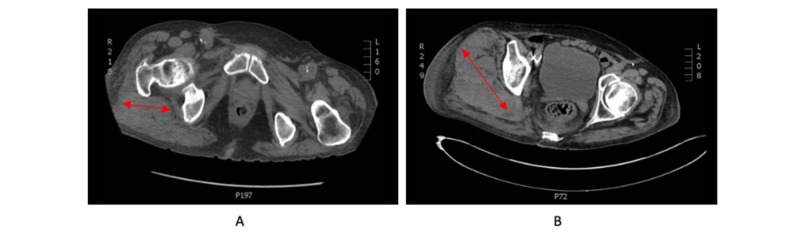
CT images of the right gluteal hematoma On initial admission, the right gluteal hematoma measured 3.8 cm x 3.1 cm (A) and on readmission, it measured 10 cm x 3.5 cm (B). CT: computed tomography

A 78-year-old man presented to the emergency department with right flank bruising and pain secondary to a mechanical fall sustained nine days prior. He was subsequently found to have a hemoglobin of 6.5 g/dL and a computed tomography (CT) scan demonstrating a right gluteal intramuscular hematoma measuring 3.1 cm x 3.8 cm (Figure 1A). The patient was known to have an extensive cardiovascular history, including multiple aortic aneurysms from the origin of the left subclavian artery to the bilateral external iliac arteries, with the largest measuring 8.5 cm x 8.2 cm in the descending thoracic aorta. Each was repaired with a thoracic aortic aneurysm graft, an abdominal aortic aneurysm graft, and bilateral common iliac grafts complicated by a type I endoleak requiring endovascular aneurysm repair, the development of a type II endoleak of the abdominal aortic aneurysm, coronary artery disease, and a cerebrovascular accident (on clopidogrel). The patient was transfused two units of packed red blood cells (pRBCs), raising his hemoglobin to 8.3 g/dL. After overnight observation, he was discharged home.

Prior to his admission, coagulation studies demonstrated low fibrinogen, elevated D-dimer, abnormal prothrombin time (PT), abnormal partial thromboplastin time (PTT), and low platelets (Table 1). The International Society on Thrombosis and Haemostasis Disseminated Intravascular Coagulation (DIC) score was five, consistent with chronic DIC. The direct antiglobulin test was negative, and there was no clinical or laboratory evidence of cirrhosis. In addition, von Willebrand disease studies were normal and mixing studies showed no evidence of an inhibitor.

**Table 1:** Coagulation profile

	Prior to admission	Before TXA therapy	After TXA therapy	Follow-up 3 weeks later (after discontinuation of TXA therapy)
Hemoglobin (g/dL)	8	7.5	8.5*	10.2
Platelets (10^3^/mm^3^)	80	77	122**	111
PT (sec)	20.3	18.7	19	17.2
PTT (sec)	37	47.3	44	37.2
Fibrinogen (mg/dL)	136	220	230	265
D-dimer (mcg/mL)	> 20	> 20	6.97	> 20
R time (min)	-	-	3.2	4.1
K time (min)	-	-	1.5	1.9
α angle (degrees)	-	-	69.4	66.4
MA (mm)	-	-	59.1	47.4
G value (kilodynes/cm^2^)	-	-	7.2	4.5
LY 30 (%)	-	-	0.7	4
* Patient received a total of 5 units of packed red blood cells				
** Patient received 1 unit of platelets				
TXA: tranexamic acid, PT: prothrombin time (reference range 12.1-14.7 sec), PTT: partial thromboplastin time (reference range 24.3-35.9), R time: reaction time (2-8 min), K time: kinetic time (1-3 min), α: alpha angle (55-78 deg), MA: maximum amplitude (51-69 mm), G value: calculated value of clot strength (4.6-10.9 kilodyne/sec), LY 30: clot lysis at 30 minutes following maximum amplitude (0-7.5%)				

Four days later, he was readmitted due to weakness and presyncope. Repeat imaging showed an interval expansion of the right gluteal hematoma, measuring now at 10 cm x 3.5 cm (Figure 1B), and his hemoglobin dropped to 6.1 g/dL. Due to severe vasculopathy, prior aneurysmal stents, and stage IV chronic kidney disease prohibiting contrast use, the embolization of the bleeding arterial vessel via intravascular access was not feasible per vascular surgery consultation.

Over the course of this second hospitalization, the patient required a transfusion of five units of packed red blood cells (RBCs) to maintain his hemoglobin > 7 g/dL. Clopidogrel was discontinued, and one unit of platelets was transfused. Hematology was consulted and an enhanced fibrinolytic type DIC was suspected. He was treated with 1 gram of intravenous TXA every eight hours on Day 5 of admission for a total of four doses. The patient’s hemoglobin stabilized after the initiation of TXA, and he required no additional transfusions (Table 1). Additionally, coagulation parameters improved with D-dimer reduction from >20 mcg/mL to a nadir of 6.97 mcg/mL and fibrinogen increased from 199 mg/dL prior to TXA to 297 mg/dL after TXA (Table 1).

The patient was discharged to home on oral TXA 1300 mg twice daily (renal dosing) for a total of five days of TXA therapy. On follow-up, exactly nine days after his last TXA dose and 13 days after his discharge from the hospital, his D-dimer was more abnormal (>20 mcg/mL) but his fibrinogen level remained in the reference range (Table 1). He had stabilized his hemoglobin, with no clinical evidence of ongoing hemorrhage and his hematoma was resolving on physical examination.

Thromboelastography (TEG) was completed after the initiation of TXA, which demonstrated no abnormalities; however, there was no baseline for comparison due to technical difficulties that prohibited a TEG study to be performed prior to the initiation of TXA. On follow-up, and after discontinuation of TXA, TEG indicated decreased maximum amplitude and decreased G value, consistent with a consumptive coagulopathy (Table 1).

## Discussion

Chronic disseminated intravascular coagulation (DIC) is an established complication of an aortic aneurysm [1-2]. It is thought that turbulent flow exposes the endothelium and tissue factor, leading to the excessive generation of thrombin, chronic consumption of coagulation factors, and excess generation of plasmin with fibrinolysis of clots. This mechanism of DIC development has also been described in type I, II, and III endoleaks following endovascular aortic repair [3-5].

The patient presented in this case report differs from other case reports in that he was not a surgical candidate, which created a therapeutic challenge. Case reports of treating DIC associated with abdominal aortic aneurysm and endoleakage with TXA have been reported [6-8]. However, to our knowledge, there have been no reports of treating a non-aortic life-threatening bleed associated with coagulopathy secondary to an aneurysm or endoleakage.

DIC can be categorized into three groups, which include DIC with suppressed fibrinolysis, DIC with enhanced fibrinolysis, and DIC with balanced fibrinolysis [9]. DIC with enhanced fibrinolysis is typically seen in patients with abdominal aortic aneurysms, and bleeding can be catastrophic. Labs in this setting typically show elevations in D-dimer, fibrin degradation products, thrombin-antithrombin complex, and plasmin-α2 plasmin inhibitor complex. In addition, the fibrin degradation product to D-dimer ratio tends to be elevated.

Although it is not clear whether our patient had DIC due to his underlying aortic aneurysms or secondary to an endoleakage, he did exhibit a consumptive coagulopathy based on his coagulation profile. In general, the best hope of correcting DIC associated with aortic aneurysms or endoleakage is with surgery [3,5]. However, the benefits of operative correction did not outweigh the potential risk of death and renal failure in our patient, who had numerous comorbidities, in conjunction with vascular surgery recommendations.

The administration of a loading dose of intravenous (IV) TXA followed by a short course of oral TXA to complete five days of therapy successfully aided in controlling the progression of this patient’s life-threatening bleed. After initiation of therapy, he no longer required additional blood products, demonstrated normalization of thromboelastography, and had a resolving hematoma on physical examination. Review of TEG three weeks after completion of therapy showed decreasing MA and G times consistent with excessive clot breakdown and a return of ongoing fibrinolysis. These abnormalities were concordant to the increased fibrin split products and his D-dimer level at this time. While baseline TEG was not immediately available prior to the administration of TXA, these values support the diagnosis of an enhanced fibrinolytic DIC and support the use of an anti-fibrinolytic in the treatment of this patient’s underlying coagulopathy.

The risk of thrombosis with TXA must be a consideration, especially in a patient with a history of cerebrovascular accident and coronary artery disease. This is especially true in the setting of enhanced fibrinolytic DIC where previous studies have demonstrated that the use of an anti-fibrinolytic can produce micro-embolisms prompting the concomitant use of anticoagulation therapy. Due to the greater concern for bleeding in our patient, systemic anticoagulation was deferred. The long-term effects, duration of therapy, and optimal dose of TXA therapy are currently unknown. The dose and duration of therapy used in our patient were based on changes in his coagulation profile and clinical evidence of resolving hemorrhage balanced with the risk of thrombosis. Additional studies need to be done to determine the endpoints of treatment with TXA (i.e. dosing to specific goals like fibrin degradation products (FDP)/D-dimer ratio). Nevertheless, TXA was successful in reversing a consumptive coagulopathy and stabilizing a life-threatening hematoma in a patient with acute hemorrhage in the setting of chronic DIC.

Following this event and the completion of the planned course of TXA, the patient demonstrated progressive coagulopathy with a rising K time, low alpha angle, low maximum amplitude (MA), and low G time, indicating reduced clot formation and strength. These values were felt to represent a progressive DIC and consumptive coagulopathy manifesting with reduced clotting factors, hypofibrinogenemia, and thrombocytopenia. He required additional doses of TXA and the use of cryoprecipitate to control subsequent bleeding episodes but ultimately developed enlarging aneurysms with a concern for the development of an aorto-enteric fistula associated with massive gastrointestinal bleeding. The patient elected for hospice and ultimately passed away more than six months from his initial medical management with oral TXA.

## Conclusions

This case report summarizes the successful use of TXA in a patient with DIC secondary to multiple aortic aneurysms and endoleakage in an effort to stop the expansion of a life-threatening gluteal hematoma not amenable to surgical or vascular intervention.
